# Supplementary material for: Direct identification of reaction sites on ferrihydrite
Source: Commun Chem. 2020 Jun 19;3:79. doi: 10.1038/s42004-020-0325-y (PMC9814833; doi:10.1038/s42004-020-0325-y)
Supplement: Supplementary file 2 — Supplementary Information [file 42004_2020_325_MOESM2_ESM.pdf]

SUPPLEMENTARY INFORMATION

Direct Identification of Reaction Sites on Ferrihydrite

Jean-François Boily* and Xiaowei Song‡

Department of Chemistry, Umeå University, SE-901 87 Umeå, Sweden

*corresponding author: jean-francois.boily@umu.se; +46 73 833 2678

‡. Current address : R&D, IKEA of Sweden AB, SE-343 34 Älmhult, Sweden

Table of Contents

1. Material Characterization 2
2. Vibration Spectroscopy 2
3. Chemometrics 3
4. Molecular Dynamics 3

Supplementary Figure 1 4
Supplementary Figure 2 5
Supplementary Figure 3 6
Supplementary Figure 4 6
Supplementary Figure 5 7
Supplementary Figure 6. 7
Supplementary Figure 7 8
Supplementary Figure 8 9
Supplementary Figure 9 9
Supplementary Table 1..10
References 11

Supplementary Methods

1. Material Characterization

Suspensions of 14.2 g/L Fh were stored in N_2(g)-filled polyethylene container at 4 ºC. A portion of the resulting washed solids was dried under a stream N_2(g) at room temperature and used for phase and chemical characterisation (Fig. S1). X-ray diffraction (Bruker d8 Advance working in θ–θ mode with Cu Kα radiation) on the dry solids confirmed that 6-line Fh was the sole crystallographic phase in the precipitates. Vibration spectra showed no evidence for the conversion of Fh to (low crystallinity) FeOOH, as can be confirmed by the lack Fe-O-H bending modes of goethite and lepidocrocite.[1] Samples imaged by transmission electron microscopy imaging (JE-1230, JEOL) revealed particle aggregates of the order of ~20-30 nm. A 90-point adsorption/desorption isotherm (TriStar, Micromeritics) on samples previously dried *in situ* at 100°C for 16 h under a stream of N_2(g) revealed a Brunauer-Emmet-Teller[2] specific surface area of 209 m^2/g and 5.5% in microporosity. These measurements suggest individual particles as small as ~7-8 nm in diameter.

We used X-ray photoelectron spectroscopy to analyse the near-surface atomic composition of Fh exposed to a vacuum of less than 10^{-7} Pa. The Kratos Axis Ultra electron spectrometer used for these measurements was equipped with a delay line detector, a monochromated Al Kα source operated at 150 W, a charge neutralizer, and a hybrid lens system with a magnetic lens providing an analysis area of 0.3 mm × 0.7 mm. We collected survey spectra from 1100 to 0 eV at a pass energy of 160 eV, while the high-resolution spectra (pass energy 20 eV) for Fe 2p, O 1s, C 1s and Cl 2p were collected at a rate of 0.1 eV/step. Using the spectra processing software of Kratos, we applied a Shirley background to all high-resolution spectra, and adjusted the binding energy scale to the 285.0 eV C 1s line of aliphatic carbon. We modeled these resulting background-subtracted spectra with a 70% Gauss/30% Lorentz function (Table S1).

2. Vibration Spectroscopy

Aqueous suspensions of Fh (14.2 g/L; 2968 m^2/L) were prepared at total concentrations of 31 NaOH/nm^2 to 81 HCl/nm^2, keeping the pH in the 2-10 range. These loadings were achieved by additional of standardised NaOH or HCl to the aqueous suspensions. All suspensions were equilibrated in sealed polyethylene test tubes for 24 h under N_2(g), then exposed to a stream of N_2 (g) for another 0.5 h prior centrifugation. The pastes were then applied onto an Attenuated Total Reflectance accessory (Golden Gate, single-bounce diamond cell), using a Bruker Vertex 70/V Fourier Transform Infrared spectrometer equipped with a DTGS detector. Measurements were carried out in the 600-4500 cm^{-1} range at a resolution of 2.5 cm^{-1} and at a forward/reverse scanning rate of 10 Hz, resulting in 1000 co-added spectra for each sample. Blackman-Harris 3-term apodisation function was used to correct phase resolution.

Temperature programmed desorption experiments were performed in an optical chamber (AABSPEC #2000-A) equipped with CaF_2 windows, and holding a vacuum of less than 0.3 Pa. Spectra were collected in the 1100-4500 cm^{-1} range at a resolution of 4 cm^{-1}. Each spectrum was the average of 50 scans.

3. Chemometrics

We used the multivariate curve resolution method [3] to facilitate interpretation of the changes in the vibration spectra in the temperature programmed desorption experiments. The method extracts spectral components (**ε**), and their respective concentration profiles (**C**) from a 2D matrix of spectral absorbances ($\mathbf{A}_{m\times n}$ of m wavenumbers and n temperatures) through the Beer-Lambert relationship $\mathbf{A} = \boldsymbol{\varepsilon}\cdot\mathbf{C}$. These spectral components are thus akin to molar absorption coefficients of relatively pure chemical species, scaled for an undetermined optical path length. In order to execute this procedure, we first offset all spectra in the O-H stretching region to zero absorbance at 4000 cm^{-1}, where absorbances from the sample are negligible. We then determined the number of chemically significant species responsible for the variance of these spectra using the Malinowski's factor indicator function[4] and starting from abstract eigenvectors and eigenvalues obtained from a singular value decomposition. [5] We then used the MCR-ALS [3] program to extract **ε** and **C** using our chosen number of species. The program rotates those number of eigenvectors of the **A** matrix into a real chemical space, such that such $\mathbf{A}_{m\times n} = \boldsymbol{\varepsilon}_{m\times s}\cdot C_{s\times n} + \mathbf{E}$, where $\boldsymbol{\varepsilon}_{m\times s} \geq 0$ and $\mathbf{C}_{s\times n} \geq 0$, and **E** is the matrix of unaccounted residuals. These calculations were made in the computational environment of MATLAB 9.1. [6]

4. Molecular Dynamics

Molecular dynamics simulations of the Fh nanoparticles were performed with program GROMACS/2019.1. [7] Simulations were carried out using the Clayff force field [8] and the revised parameter for iron.[9] Following the practice of modelling tetrahedrally coordinated metal ions in clays, we treated the tetrahedral Fe3 site with the same interaction potentials values as the octahedral iron but constrained its O-Fe3-O angle of 109.5°. A NVT (constant number of particles, constant volume and constant temperature) ensemble and a time step of 1.0 fs were used with the Verlet algorithm [10] to integrate the equations of motions for all the atoms in the system, which were projected using a periodic boundary condition. The temperature of the system (300 K) was coupled to the Nosé-Hoover [11] velocity-rescale thermostat with a 0.1 ps relaxation time. The O-H bond strength of all the hydroxyls were treated by the LINCS[12] algorithm. A 0.8 nm cutoff was used for non-bonded van der Waals interactions and the particle-mesh Ewald [13] method was used to treat long-range electrostatic interactions.

Simulations cells were first energy-minimised (double precision) using a steepest descent algorithm. The resulting structure was then equilibrated (single precision) using classical MD for at least 10^7 steps (10 ns), followed by production runs of at least another 10 ns. Total energy convergence and its components as well as temperature, and atomic densities were monitored for these entire equilibration periods. This modelling strategy retained the Fh bulk structure, yet relaxed the surface. Radial distribution functions for atomic pairs and hydrogen bond analyses were carried out using the utilities of GROMACS/2019.1. [7] Radial distribution functions with respect to the particle center of mass were however generated using a MATALB 9.1 [6] code written for this work.

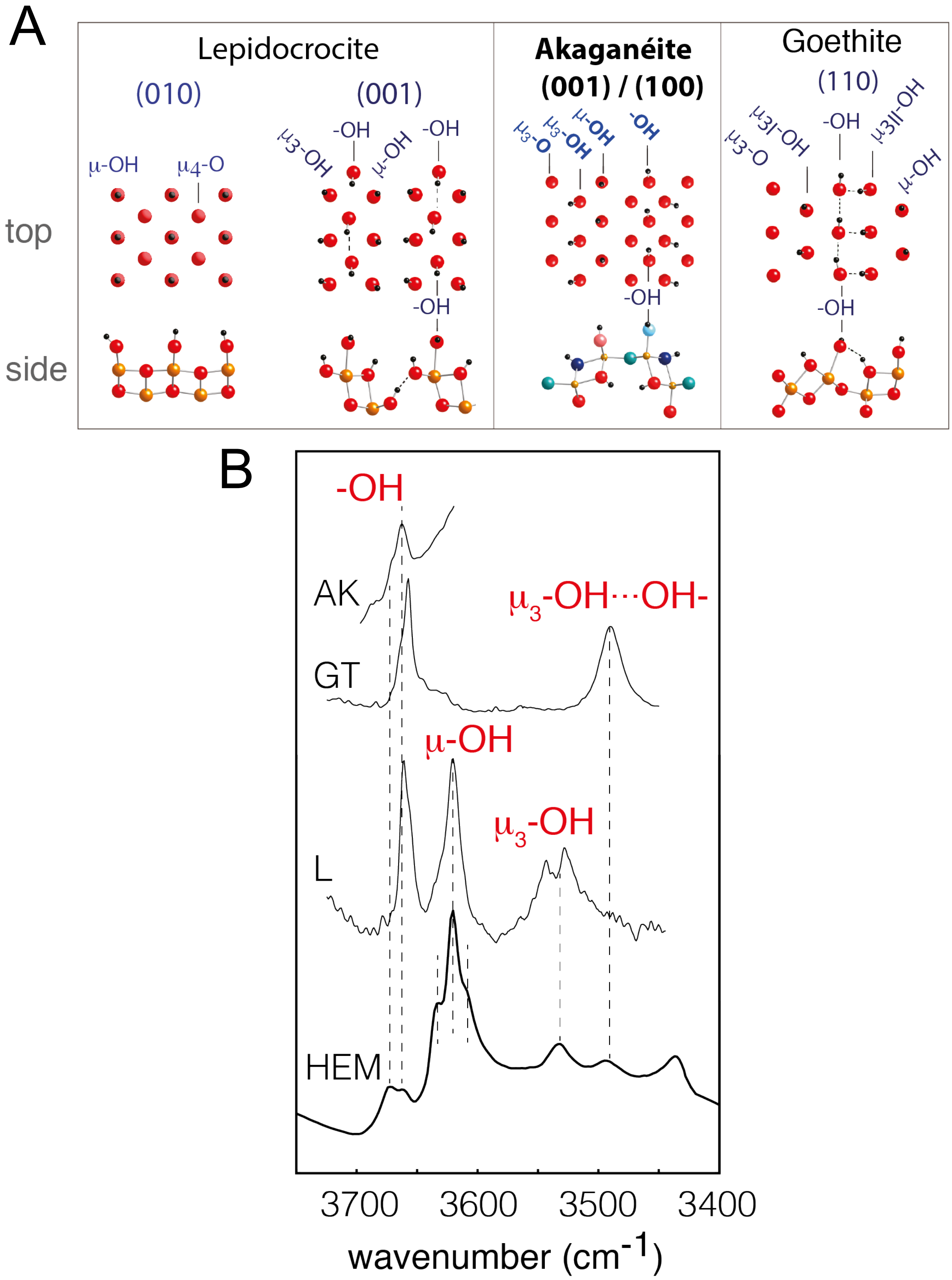

Supplementary Figure 1. Reference spectra on crystalline iron (oxy)(hydro)oxides revealing surface OH populations. (A) Representative OH populations of dominant crystallographic faces of FeOOH mineral. (B) Reference O-H stretching spectral signatures of OH groups. (AK=akaganéite (β-FeOOH); GT=goethite (α-FeOOH); L=lepidocrocite (γ-FeOOH); HEM=hematite (α-Fe_2O_3)). All data were extracted from previous studies from our group.[14-16]

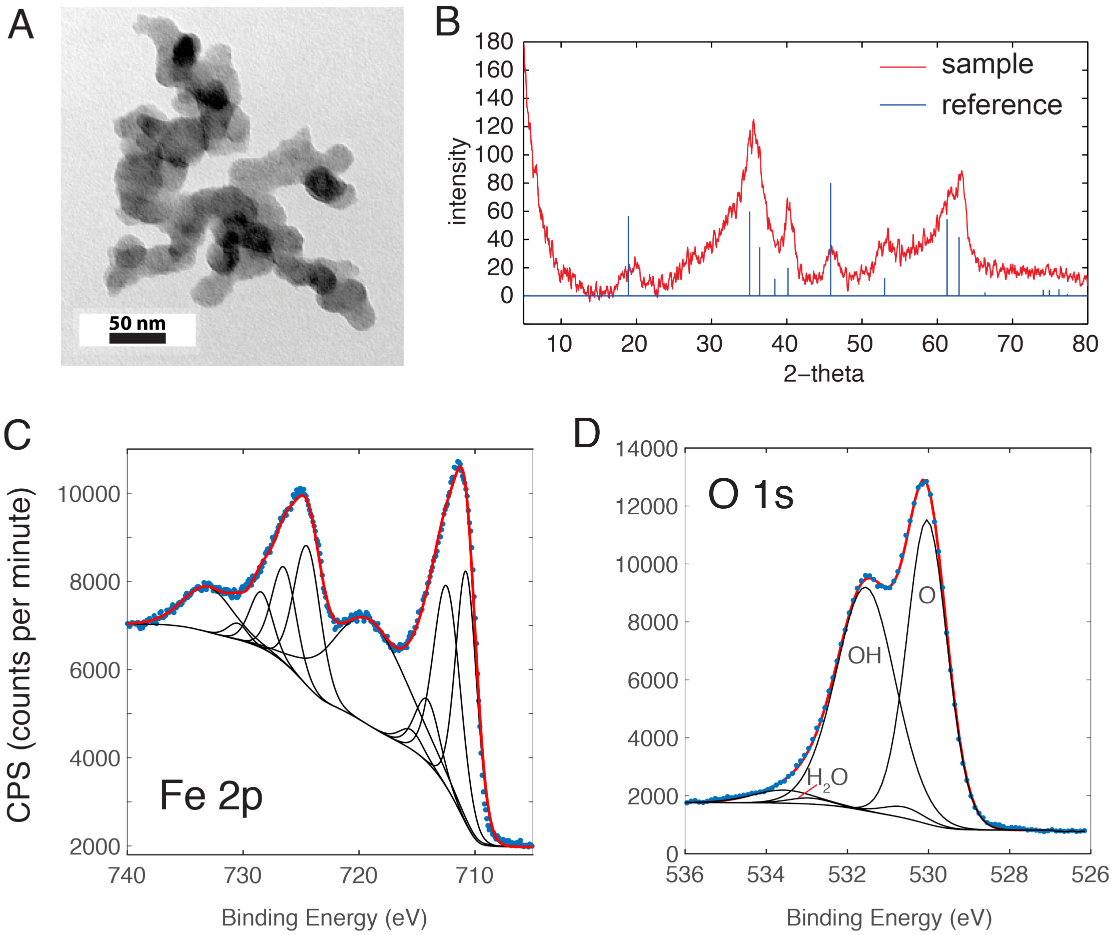

Supplementary Figure 2. Ferrihydrite characterization. (A) Transmission electron microscopy spheroidal Fh particles and/or aggregates of the order of ~20-30 nm (Fig. 1A). Individual particles could however be as small as ~7-8 nm in diameter given their B.E.T. specific surface area of 209 m^2/g. (B) XRD pattern of the 6-line ferrihydrite sample, and compared against PDF #00-058-0900 ($Fe_{9.5}O_{14}(OH)_2$). (C-D) X-ray photoelectron spectroscopy of the (C) Fe 2p and (D) the O1s regions, including fits. The O1S region reveals an OH/O ratio of 1.1. XPS spectra were shifted to the C1s peak at 285.0 eV.

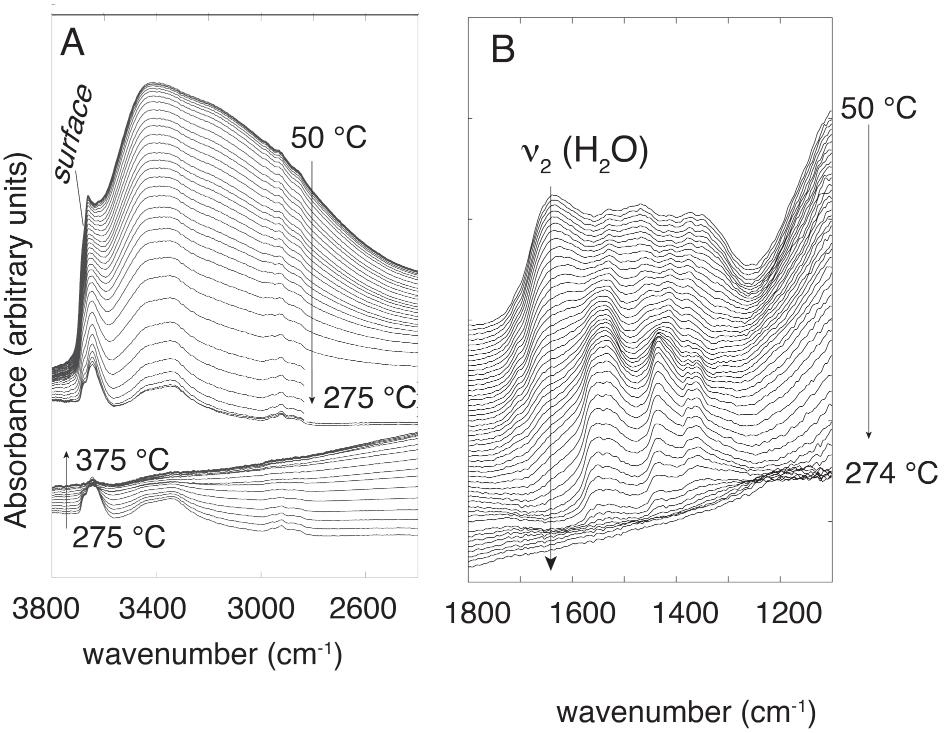

Supplementary Figure 3. Temperature-programmed desorption of Fh *in vacuo* showing the broad (A) O-H stretching from 50 to 375 °C, and (B) water bending band at ~1630 cm^{-1} confirming the resilience of Fh-bound water to temperature.

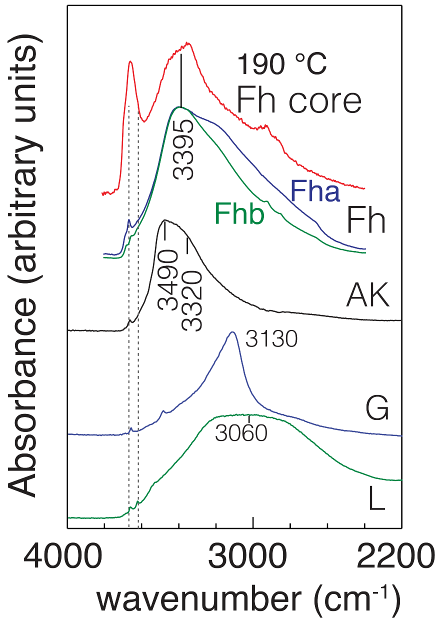

Supplementary Figure 4. Spectral signature of a Fh core taken *in vacuo* at 190 °C (Fig. 3 of main text) compared to those of Fh and other reference FeOOH minerals at 25 °C. (AK=akaganéite (β-FeOOH); GT=goethite (α-FeOOH); L=lepidocrocite (γ-FeOOH);). All data were extracted from previous studies[14-16] from our group.

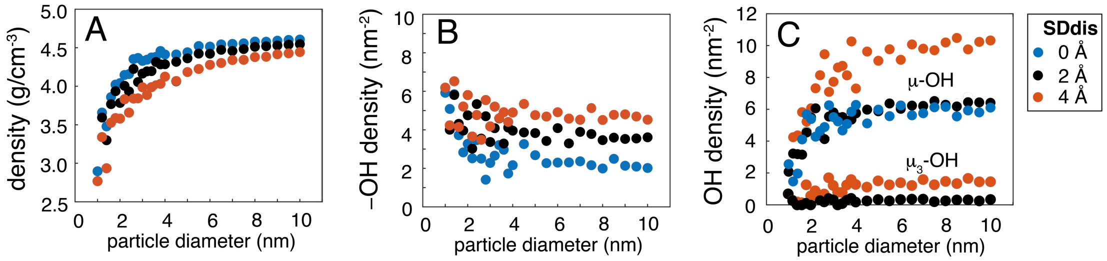

Supplementary Figure 5. Fh nanoparticle simulations at selected diameters, and surface depletion depths of SDdis 0, 2, and 4 Å. (A) Particle mass densities, (B) –OH site densities, and (C) μ–OH and μ_3–OH site densities. Simulations of SDdis = 6 Å give identical densities for –OH groups (not shown). All particles were charge-neutral.

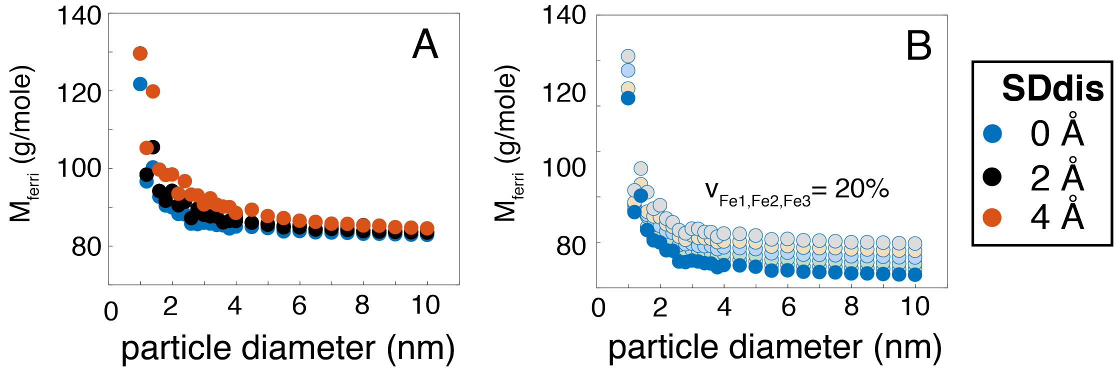

Supplementary Figure 6. Simulated molar masses of Fh at (A) various surface depletion depths (SDdis) and (B) Fe vacancies (v_{Fe1},v_{Fe2},v_{Fe3}).

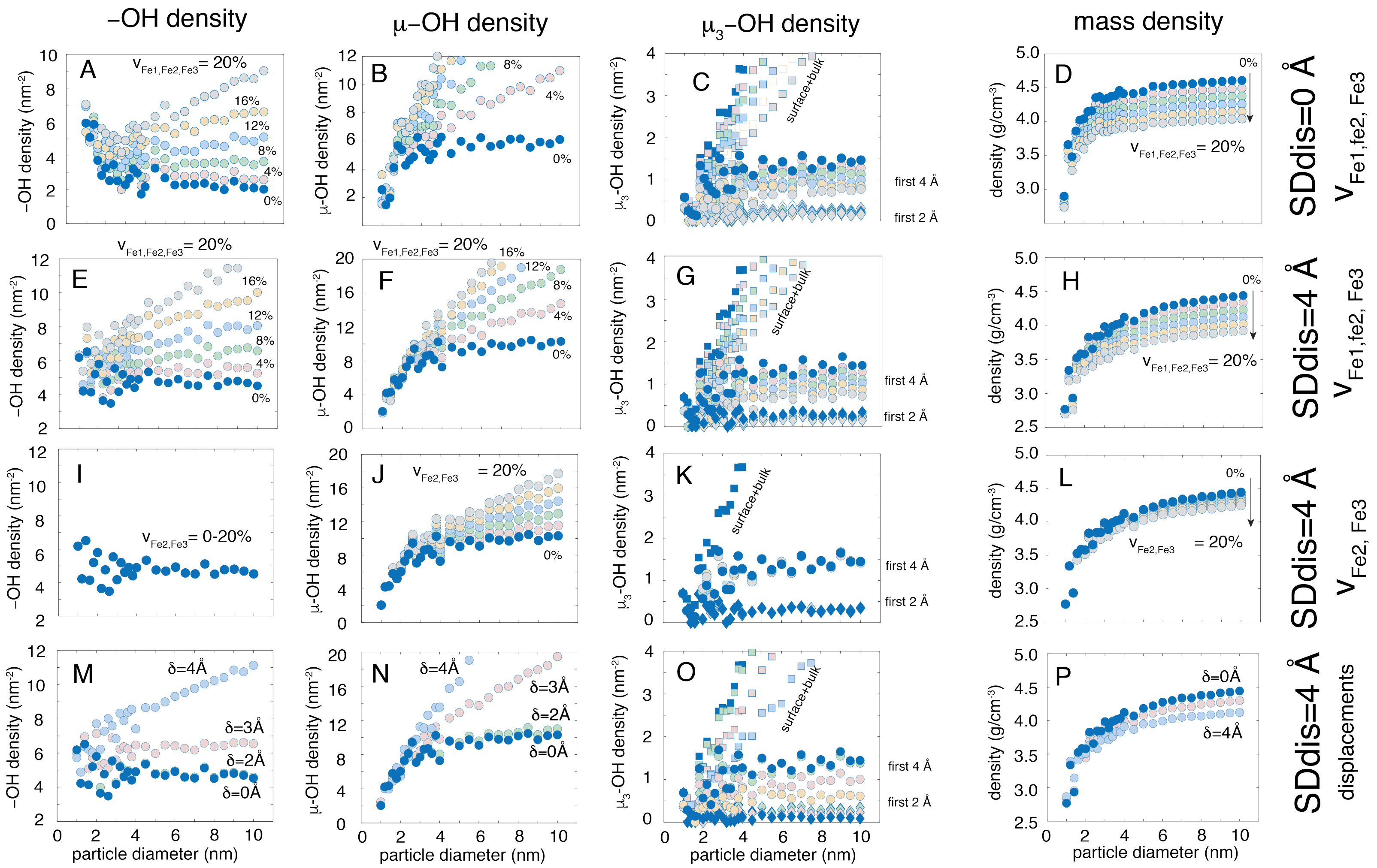

Supplementary Figure 7 Fh nanoparticle simulations at selected diameters, and surface depletion depths (SDdis=0-4 Å), Fe vacancies ($v_{Fe1,Fe2,Fe3}$ for all Fe (1rst and 2nd rows) and $v_{Fe2,Fe3}$ for Fe2 and Fe3 sites only (3rd row)), and atomic displacements (4th row). –OH (A,E,I,M), µ–OH (B,F,J,N), µ3–OH (C,G,K,O) and mass (D,H,L,P) densities. All particles were charge-neutral.

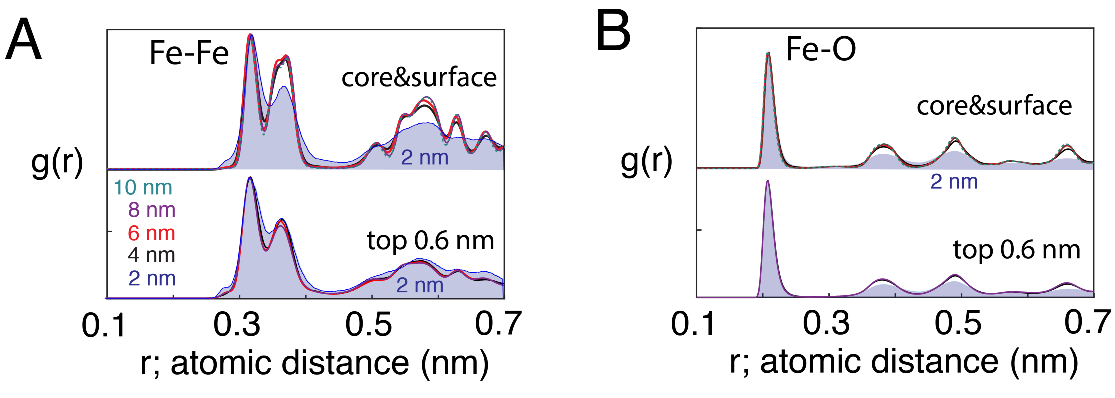

Supplementary Figure 8. Radial distribution function (RDF) for (A) Fe-Fe and (B) Fe-O atomic distances. Here we compare the values for all atoms (core&surface, as in Fig. 5C of main text) with those for only the top 0.6 nm of the particles. These results show that the RDF values he combined core and surface of the 2 nm particles are dominated by the surface contributions, while those of the larger-sized particles are dominated by the core. Generated by Molecular Dynamics simulations of single Fh nanoparticles with diameters of 10, 20, 30 and 40 nm. –OH group are bound to only

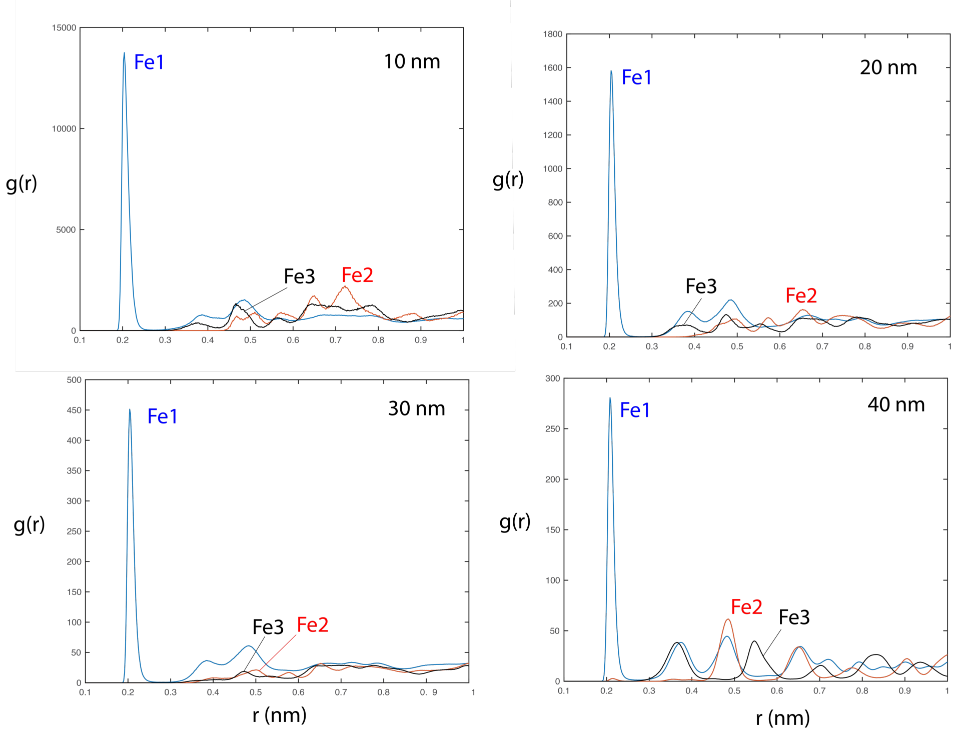

Fe1.

Supplementary Figure 9. Radial distribution function of atomic distances between Fe (Fe1, Fe2, Fe3) and –OH groups of the Fh surface. Generated by Molecular Dynamics simulations of single Fh nanoparticles with diameters of 10, 20, 30 and 40 nm. –OH group are bound to only Fe1.

Supplementary Table 1 **X-ray photoelectron spectroscopic analysis of Fh (Fig. S1 C-D)**

Name	Position (eV)	FWHM (eV)	Line Shape	R.S.F.[a]	% Conc. (At.%)
Fe 2p 3/2 1	710.74	2.15	GL(30)	2.957	6.118
Fe 2p 3/2 2	712.41	2.727	GL(30)	2.957	6.189
Fe 2p sat 1	714.1	2.495	GL(30)	2.957	1.773
Fe 2p sat 2	715.5	2.3	GL(30)	2.957	0.501
Fe 2p sat 3	719.11	7.574	GL(30)	2.957	9.009
Fe 2p 1/2 1	724.47	2.687	GL(30)	2.957	4.208
Fe 2p 1/2 2	726.47	2.486	GL(30)	2.957	2.663
Fe 2p sat 4	728.44	2.328	GL(30)	2.957	1.458
Fe 2p sat 5	730.47	2.02	GL(30)	2.957	0.369
Fe 2p sat 6	733.01	4.998	GL(30)	2.957	2.517
O 1s O	530.03	1.171	GL(30)	0.78	24.611
O 1s OH	531.54	1.718	GL(30)	0.78	26.542
O 1s H_2O	533.56	1.8	GL(30)	0.78	1.624
O 1s COC[b]	532.92	1.2	GL(30)	0.78	0.493
O 1s COC=O[b]	530.6	1.2	GL(30)	0.78	0.919
C 1s CH[b]	285	1.318	GL(30)	0.278	4.702
C 1s COC[b]	286.21	1.8	GL(30)	0.278	2.963
C 1s COC=O[b]	288.83	1.794	GL(30)	0.278	2.756
Cl 2p 3/2[c]	198.62	1.241	GL(30)	0.891	0.39
Cl 2p 1/2[c]	200.22	1.241	GL(30)	0.891	0.195
**Name**	**At. ratio**			**total % Conc. (At.%)**	
Fe (total)				34.805	
O 1s O				24.611	
O 1s OH				26.542	
O 1s H_2O				1.624	
O/Fe	0.71				
OH/Fe	0.76				
OH/O	1.08				
H_2O/Fe	0.05				

a. Sensitivity factor;
b. n.b. inorganic and organic carbon contaminants originate from the atmosphere, and acquired during adventitious exposure to ambient air.
c. Trace chloride contaminants are remnants from the synthesis procedure.

Supplementary References

1 Song, X. & Boily, J.-F. Surface and Bulk Thermal Dehydroxylation of FeOOH Polymorphs. *J. Phys. Chem. A* **120**, 6249-6257.

2 Brunauer, S., Emmet, P. H. & Teller, A. Adsorption of gases in multimolecular layers. *J. Am. Chem. Soc.* **60**, 309-319.

3 Jaumot, J., Gargallo, R., de Juan, A. & Tauler, R. A graphical user-friendly interface for MCR-ALS: a new tool for multivariate curve resolution in MATLAB. *Chemometrics and Intelligent Laboratory Systems* **76**, 101-110.

4 Malinowski, E. R. *Factor Analysis in Chemistry, 3rd Edition*. (Wiley-VCH, 2002).

5 Golub, G. H. & Reinsch, C. Singular value decomposition and least squares solutions. *Numerische Mathematik* **14**, 403-420.

6 version 9.7.0 (R2019b) (Natick, Massachussets: The MathWorks Inc., 2019).

7 Van Der Spoel, D. *et al.* GROMACS: Fast, Flexible, and Free. *Journal of Computational Chemistry* **26**, 1701-1718.

8 Cygan, R. T., Liang, J. J. & Kalinichev, A. G. Molecular models of hydroxide, oxyhydroxide, and clay phases and the development of a general force field. *J. Chem. Phys. B.* **108**, 1255-1266.

9 Kerisit, S. Water structure at hematite–water interfaces. *Geochimica et Cosmochimica Acta* **75**, 2043-2061.

10 Hockney, R., Goel, S. & Eastwood, J. Quiet High-Resolution Computer Models of a Plasma. *Journal of Computational Physics* **14**, 148-158.

11 Nose, S. & Klein, M. L. Constant Pressure Molecular-Dynamics for Molecular-Systems. *Molecular Physics* **50**, 1055-1076.

12 Hess, B., Bekker, H., Berendsen, H. J. & Fraaije, J. G. LINCS: A Linear Constraint Solver for Molecular Simulations. *J. Comp. Chem.* **18**, 1463-1472.

13 Darden, T., York, D. & Pedersen, L. Particle Mesh Ewald: An N.log (N) Method for Ewald Sums in Large Systems. *J. Chem. Phys.* **98**, 10089-10092.

14 Boily, J. F. *et al.* Thin Water Films at Multifaceted Hematite Particle Surfaces. *Langmuir* **31**, 13127-13137.

15 Song, X. & Boily, J. F. Structural controls on OH site availability and reactivity at iron oxyhydroxide particle surfaces. *Phys. Chem. Chem. Phys.* **14**, 2579-2586.

16 Song, X. & Boily, J. F. Surface Hydroxyl Identity and Reactivity in Akaganeite. *J. Phys. Chem. C* **115**, 17036-17045.
